# Overview of neurosarcoidosis: recent advances

**DOI:** 10.1007/s00415-014-7482-9

**Published:** 2014-09-07

**Authors:** Renata Hebel, Mirosława Dubaniewicz-Wybieralska, Anna Dubaniewicz

**Affiliations:** 1 Department of Neurology, Medical University of Gdansk, Debinki 7 Str, 80-211 Gdansk, Poland; 2 Department of Radiology, Medical University of Gdansk, Debinki 7 Str, 80-211 Gdansk, Poland; 3 Department of Pneumology, Medical University of Gdansk, Debinki 7 Str, 80-211 Gdansk, Poland

**Keywords:** Neurosarcoidosis, Neurosarcoidosis forms, Clinical symptoms, Diagnosis, Treatment

## Abstract

Sarcoidosis (SA) is a granulomatous, multisystem disease of unknown etiology. Most often the disease affects lungs and mediastinal lymph nodes, but it may occur in other organs. Neurosarcoidosis (NS) more commonly occurs with other sarcoidosis forms, in 1 % of cases it involves only nervous system. Symptomatic NS occurs but on autopsy study up to 25 % of cases are confirmed. NS can affect central nervous system: the brain, spinal cord and peripheral nerves, and muscles. The diagnosis of neurosarcoidosis facilitates diagnostic criteria: histopathological, imaging and cerebrospinal fluid examination, and clinical symptoms. At present, there are no set standards for treatment of patients suffering from NS. Early therapy of symptomatic patients is recommended. Corticosteroids still are the first line of treatment for NS patients. In cases of steroids resistance, lack of their effectiveness or existence of contraindication to their use, immunosuppressant treatment is recommended. The latest NS algorithm with immunosuppressive treatment is discussed.

## Introduction

**Fig. 1:**
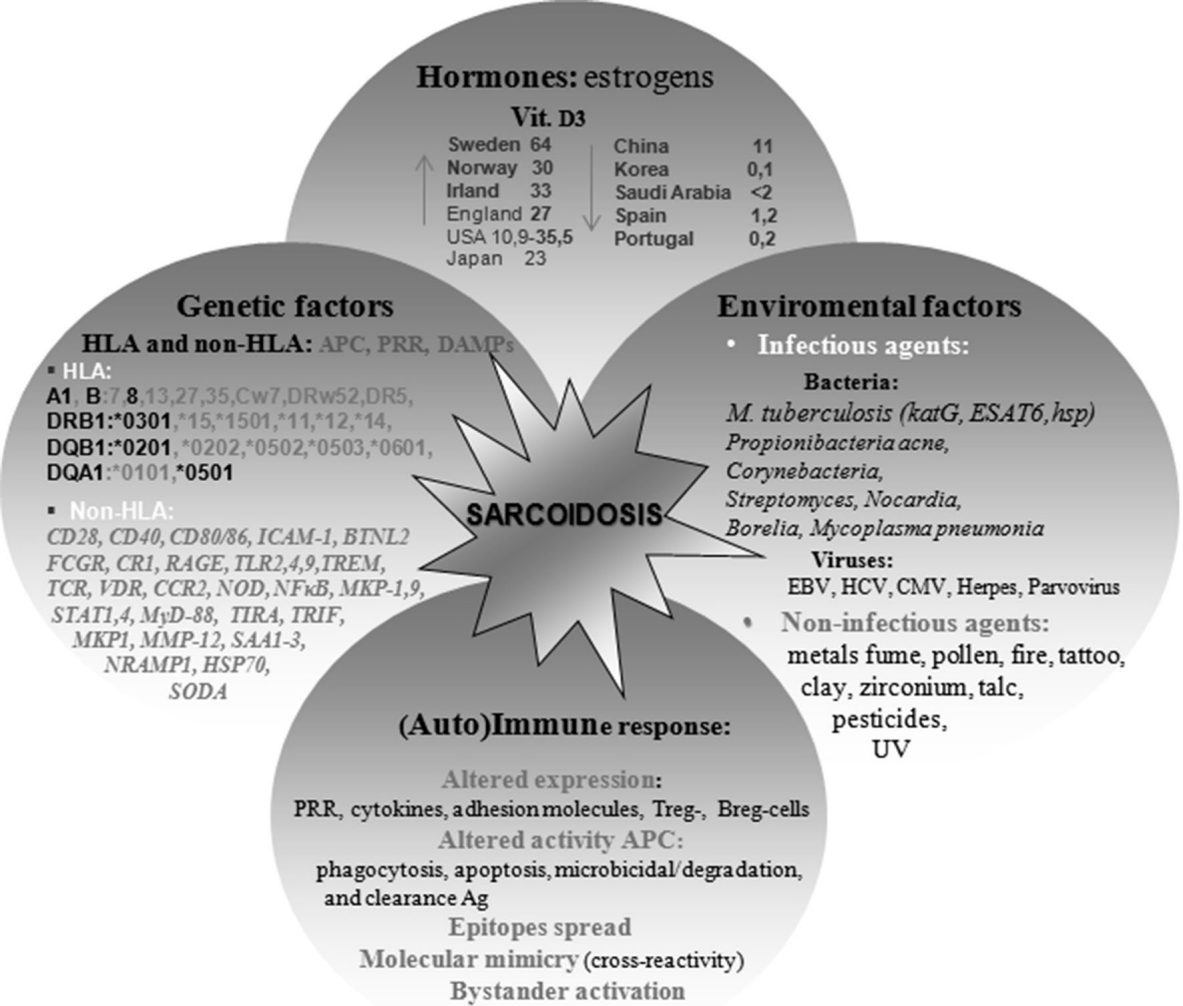
The etiopathogenesis of sarcoidosis

Sarcoidosis (SA) is a multisystem disorder of unknown cause [1–15]. In the light of the modified Matzinger’s model of immune response [8] and results of studies conducted by Dubaniewicz et al. [reviewed in 7] and other researchers [reviewed in 7, 9–15], human heat shock proteins (HSPs) as main ‘danger signals’ (tissue damage-associated molecular patterns-DAMPs) and/or microbial HSPs as pathogen-associated molecular patterns (PAMPs) recognized by pattern recognition receptors (PRR), may induce sarcoid inflammation (lymphocytes CD4 in excess of CD8 in affected organs with an insufficient number and/or function of CD4 cells within the blood) by both infectious and non-infectious factors in a genetically differently predisposed host (Fig. 1) [reviewed in 1, 7].

SA most frequently occurs in Northern Europe, Japan and in central USA and its eastern coast, particularly in adults about 30–40 years old and in older adults, especially in women (Fig. 1) [1–6]. Among Afro-Americans, SA occurs ~ 4 times more frequently than among Caucasians or Asians, and it is as a rule of a more advanced process in character. The risk of death from SA is estimated at 1–5 % and connected with respiratory insufficiency in Europe and the USA, whilst in Japan the cause of death is from the cardiac form of SA. Previous limited epidemiological data in Poland puts the rate of disease from SA at about 10 in 100,000 inhabitants [5].

Due to the unknown etiology of sarcoidosis and a lack of specific diagnostic tests the diagnosis of SA relies on clinical and radiological examinations and confirmed histo-pathological biopsy results of the organ affected by the disease process, which identify the characteristic noncaseating granuloma (in about 80 %) following the exclusion of other causes of granuloma formation, primarily tuberculosis [1–6].

Imaging is an effective method of diagnosis, such as chest X-ray (CXR), high resolution computer tomography (HRCT), magnetic resonance with gadolinium (MRI) and/or positron emission tomography with fluorodeoxyglucose (FDG-PET) [1–8, 16–18]. Less effective in diagnosis are radioisotope methods (using Tall 201, Gal67, 99 m-Tc), which show defect of perfusion, which after eliminating circulatory insufficiency disease, can be a relevant indicator in diagnosis.

The most helpful diagnostic tool in pulmonary SA has turned out to be a bronchoalveolar lavage (BAL) in the presence of cellular composition. An increase index of lymphocytes CD4/CD8 greater than 3.5 indicates an active sarcoidosis process. Some centers emphasize the high diagnostic value of increased expression of integrin CD103, a differentiating antigen in immunological cells, including in BAL fluid the CD103^+^CD4^+^/CD^+^4 ratio < 0.2, CD4/CD8 > 3, CD4^+^/CD8^+^ BALF/peripheral blood > 2 [reviewed in 1–6].

Other tests helpful in the diagnosis of SA include: an elevated concentration of calcium in both blood and urine, a complete blood count with often lymphopenia, liver function tests, increased CRP, immunoglobulin G, level of soluble receptor for IL-2 (sIL-2R) and neopterin. The high concentration of serum angiotensin-converting enzyme (ACE) present in only 24–76 % of SA patients can also be present in tuberculosis and neoplasm, therefore, the diagnostic value is limited. It is mostly useful in diagnosing sarcoidosis of the cerebrospinal meninges or in monitoring the course and treatment of this disease [19, 20]. In a few states in the USA, the Kveim-Sitzbach test is also used to diagnose SA [1].

The clinical course of SA can be symptomless, where sarcoidosis is discovered accidentally during a routine examination to diagnose some other diseases or symptoms. SA patients with an acute stage of disease often complain of symptoms connected with Löfgren`s syndrome (bilateral hilar lymphadenopathy, arthritis, erythema nodosum with/without a high temperature). In 30 % of sufferers, after 2 years it takes on the chronic form with a cough, breathlessness, and respiratory insufficiency [1–6].

The most frequent form of this disease in Europe and USA is sarcoidosis of the respiratory system, although many other forms exist (Table 1) [1–6]. Extrapulmonary sarcoidosis may coexist with pulmonary SA, can overtake it or occur following remission of the pulmonary form, sometimes after several years (Table 1) [20–33]. Therefore, all patients with sarcoidosis should have pulmonary, ophthalmic, cardiac, skin, neurological, and abdominal organs examinations as well as radiological, EKG/ECHO, and laboratory tests to detect pulmonary and/or extrapulmonary manifestations of the disease [20].

**Table 1:** The occurrence of forms of sracoidosis (SA) [reviewed in 1, 20]

Forms of sarcoidosis	Occurrence (in %)
**Pulmonary sarcoidosis**	
**Respiratory system** a possible spontaneous remission in stages I–III of SA: stage I—bilateral hilar lymphadenopathy, stage II—lymph-adenopathy and diffuse pulmonary infiltrations, stage III—diffuse pulmonary infiltrations with fibrosis only in lung parenchyma, stage IV—irreversible fibro-cavernous changes in the lungs; bronchial mucosae (30–60 %); alveolar form (snowballs) (1.5 %)	90
**Pleura** (bi)lateral fluid, fibers	0.7–10
**Extrapulmonary sarcoidosis**	
**Nervous system**	5–15—symptomatic, 25—on autopsy
**Heart** arrhythmia, sudden death (SA in ventricular septum often affects the cardiac conduction system), myocarditis, pericarditis with/without pericardial effusion, mitral regurgitation, congestive heart failure, myocardial scarring with the formation of ventricular aneurysms	5—symptomatic, 25—on autopsy
**Eye(s)/orbital** anterior uveitis (50–90 %), intermediate uveitis, posterior uveitis with retinal perivasculitis, periphlebitis, neovascularization, vitreous hemorrhage, proliferative retinopathy, conjunctivitis, orbital mass lesions, extraocular myopathy, cornea involvement, optic neuropathy with disturbed vision or vision loss	25–90
**Liver/spleen**	<65
**Bone/joint** lytic/sclerotic changes in bone (e.g. skull, nasal bones, vertebrae), jungling’s syndrome (cystic spreading of the short bones of the hand and foot), acute and chronic arthritis	5–40
**Skin** erythema nodosum, lupus pernio, macules, papules, sub-cutaneous nodules, plaques, ichthyosis, ulcers, pustules, alopecia, in post-operative scars, in tattoos, erythroderma, hypopigmented patches	25–35
**Peripheral lymphadenopathy**	30
**Calcium metabolism/kidneys** hipercalcemia, hipercalciuria, nephrolithiasis	2–63
**Lacrimal glands** keratoconjunctivitis sicca syndrome, proptosis	15–28, but up to 88 % on Gal67 scanning
**Parotid glands** Heerfordt’s syndrome (fever, uveitis, inflammation of lacrimal and parotid glands, paresis n. VII), Mikulicz’s syndrome (fever, uveitis, inflammation of lacrimal and parotid glands)	5
**Reproductive tract**	<4
**Digestive system**	<1
**Muscles**	<1

To life-threatening forms of extrapulmonary sarcoidosis belong ocular SA, cardiac SA, and the nervous system SA.

## Sarcoidosis of the nervous system (neurosarcoidosis—NS)

The first case of neurosarcoidosis was described by Winkler in 1905 [reviewed in 28]. Similarly, as in cardiac sarcoidosis, NS appears symptomatically in about 5–10 % of cases, but up to 25 % are indicated from postmortem [6, 20–33]. A case control etiologic study of sarcoidosis (ACCESS) showed that neurosarcoidosis was more common in females than in males (6.0 vs. 2.2 %) [6]. The usual mean age of onset is from 33 to 41 years, slightly later compared to other forms of SA [reviewed in 30].

Neurosarcoidosis can appear with other forms of SA, e.g. pulmonary (in about 88–94 %), ocular SA (in 37–55 %) and SA of the skin (in 30 %), or it can be isolated, in about 1 % and limited only to the nervous system [25]. The course of NS is most frequently acute or subacute and in about 30 % of cases, leads to the chronic phase [27].

NS may also affect the central nervous system: the brain, spinal cord and peripheral nerves and muscles [6, 20–33]. Early NS manifestation often presents with sarcoid granuloma in the central nervous system and later may affect the peripheral nervous system and muscles [22]. Considerable diagnostic problems are caused by isolated, disseminated forms as well as symptomless or subclinical NS. Neurosarcoidosis also coexists with other neurological diseases e.g. demyelinating processes, vascular disorders or neuroborreliosis frequently making diagnosis difficult [21, 25, 33, 34]. Presently two sets of criteria have been proposed for identifying NS. In accordance with Zajicek [29], neurosarcoidosis can be defined as follows:

- Confirmed NS—the clinical picture suggests NS, other causes of neurological symptoms have been excluded, there is a histopathological presence of characteristic granuloma in the biopsy material in the central nervous system.
- Probable NS—the clinical picture suggests NS, other causes of neurological symptoms have been excluded, changes noted in an MRI scan indicate the presence of NS, the presence of an increased level of protein and/or pleocytosis particular lymphocytic, as well as the presence of oligoclonal bands confirmed in the cerebrospinal fluid, where the presence of systemic sarcoidosis has been confirmed histopathologically, or there have been at least 2 imaging results (Gal67 scintigraphy, CXR), there is an increased level of ACE in serum.
- Possible NS—the clinical picture suggests NS and other causes of neurological symptoms have been excluded.

According to Judson et al. [6], the criteria are as follows:

- Confirmed NS—the patient suffering SA presents symptoms of diabetes insipidus, paresis of the facial nerve, an MRI head scan (with gadolinium contrast) confirms changes in the cerebrospinal meninges or the brain stem, in the cerebrospinal fluid are increased levels of cells, particularly, lymphocytes and/or increased protein, a biopsy of nerve tissue reveals granulomas inflammation.
- Probable NS—neuropathy of undefined cause appears in the patient, MRI confirms abnormalities other than those defined above, electrodiagnostic tests show abnormal results.
- Possible NS—a patient suffering multiorgan SA presents with unexplained headaches and/or radiculopathy.

Recently, Marangoni et al. [35] proposed some modification to the proposals of Zajicek [29]. Instead of the traditional CXR, they suggest HRTC and instead of measuring of ACE level in serum, they propose an evaluation of the CD4/CD8 T cell ratio > 3.5 in BALF and CD4/CD8 > 5 in the cerebrospinal fluid.

## Sarcoidosis of the central nervous system (CNS)

Aggressive and/or scattered sarcoid changes in the CNS appear as small or large nodules and can be the cause of neurological symptoms, depending on their location [26]. NS can most often appear in the cerebral meninges, especially leptomeninges, with a tendency to localize at the base of the brain, in white matter, intraventricular epithelium, chorioidea, and vessel walls supplying the nervous system [27]. Supratentoral sarcoid granulomas are more frequent than subtentoral [26].

## Cerebral NS

In adults suffering from NS, the rates of sarcoid inflammation in the specific parts of the brain are as follows:

- Cranial neuropathy (brain base)—in about 23–73 % [30]
  - Paresis of n. VII—usually one-side (in 25–50 %), rarely both sides
  - Damage of n. II—blurred vision, abnormal color recognition, disturbed field of vision, papilledema in the fundus of the eye (in 1–10 %)
  - Damage of n. VIII—deafness, dizziness (in 7 %)
  - Damage of n. IX and X—dysfunction of the throat muscles, palate and vocal chords (in 4 %).
- Brain tumors (effect of mass)—in about 35–50 % [30, 32]
- Disseminated changes—in 30 % [32]
- Aseptic meningitis—in 8–40 % [30]
- Endocrinal disturbance of the hypothalamus and hypophysis in the course of NS (in 2–26 %) with primarily diabetes insipidus, polydipsia and polyuria, galactorrhoea, and amenorrhoea [30]

Cerebral NS is characterized by its wide spectrum of nonspecific general symptoms such as headache, fatigue, dizziness, fever or subfever. NS can also be accompanied by mood swings such as euphoria or depression, disturbed behavior e.g. aggression, apathy or symptoms of dementia, hallucinations or delusions [6, 20–33]. In clinical tests of those patients however, the most frequent symptom is isolated cranial neuropathy, which usually has an aggressive course in contrast to multi neural neuropathy of chronic course. Both types are characterized by good prognosis [23, 24]. In 5–38 %, NS can be a cause of (non) communicative hydrocephalus, which is usually chronic with very poor prognosis and may be the cause of death in up to 75 % of cases. Epileptic seizures generalized and partial and simple or complex appear in up to 15 % of patients with NS and can be connected with poor prognosis of the disease [21, 36]. However, the rates are different among children. Epileptic seizures occur and symptoms connected with the effect of cerebral NS predominantly occur, whilst cranial neuropathy is rare [37].

**Fig. 2:**
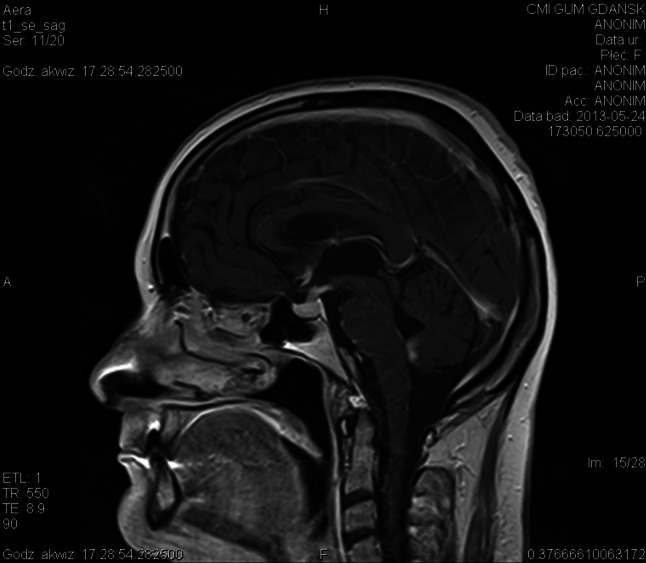
Neurosarcoidosis in MRI brain in sagital plane: T1-weighted contrast-enhanced MR image shows basal leptomeningeal enhancement and an extensive enhancement of the pituitary gland and stalk, which is markedly enlarged
  - Gal67 scintigraphy—in 2/3 of patients changes can be revealed, however, in the majority they are unspecific; one characteristic image in scintigraphy, but also in FDG-PET, observed in sarcoidosis of the parotid and lacrimal glands is called a “panda sign” [45].
  - FDG-PET—allows for a localization of changes in whole body and finding a characteristic intrathoracic pattern and/or “panda sign” may be highly suggestive for sarcoidosis; isolated NS needs additional diagnostic tests, because FDG-PET does not distinguish the changes in the course of malignancy or TB from NS [16, 40–47]; recently, it was revealed that a combination multiple fluorodeoxyglucose PET-avid lymph nodes with mild flurothymidine (FLT) PET uptake can be helpful in differentiating granulomatous inflammatory diseases such neurosarcoidosis from malignancy [42]; FDG-PET and FLT-PET are more useful methods in localizing the optimum site for a biopsy than Gal67 scintigraphy and MRI [37, 42].
- Neurophysiological tests—the effectiveness of a visual evoked potentials (VEPs) test is recommended particularly, as an affected optic nerve can be symptomatic of NS, and also auditory evoked potentials (AEPs) with abnormal results suggested NS changes in the brain stem, precursory subclinical or clinical manifestations; equally VEP and AEPs are useful in monitoring the progress of the disease [21, 25]; the advantage of such tests are that they are noninvasive, do not require prior preparation of the patient, are simple to carry out and can be repeated.
- A biopsy of the cerebrospinal meninges or brain remains the gold standard for the diagnosis of NS; in the case of negative results of the biopsy or the existence of contraindication to its use, a tissue diagnosis of sarcoidosis can often be established in an extraneural location, e.g. biopsy of lymph nodes, lungs, conjunctiva or lacrimal/parotid glands (Table 1) [6, 20–33].

Useful in the diagnosis of cerebral NS are:

- Cerebro-spinal fluid examination—changes are frequent (in about 2/3 cases) but unspecific and appear mainly in cranial neuropathy or meningitis [38]; there is often mild or moderate lymphocytosis with increased index of lymphocytes C4/CD8 > 5, increased protein concentration, a hypoglycorrhachia with CSF glucose less than 50 % of concomitant blood glucose (in 10–20 %), increased level of ACE (in about 50 %), increased IgG level, and uncharacteristic oligoclonal bands (in about 30 %) [19, 21, 34, 37]; pleocytosis and hypoglycemia often appear in the acute phase of the disease [34]; in NS, patients with an isolated cranial neuropathy of nerve VII, cerebrospinal fluid is mostly normal [37].
- Imaging methods (CT, MRI brain scan with gadolinium, Gal67 scintigraphy and FDG-PET) [18, 20, 30, 35, 39–47]
  - MRI—is considered the most sensitive but unspecific test in diagnosis patients with NS (Fig. 2) [23, 36, 38, 39, 45]; the most common brain MRI finding of NS is a basilar leptomeningeal involvement (in about 30–40 %), which is usually occurred as a thickening and diffuse or nodular enhancement; brain NS in MRI can be also present as focal masses or diffuse thickening of dural meninges, persistent pseudotumor changes (in about 14 %); involvement of hypothalamus/pituitary and cranial nerves, which show enhancement and thickening post gadolinium; rarely NS changes in MRI occur as hydrocephalus or in the periventricular region and in the white matter.

## Sarcoidosis of the spinal cord

The spinal form of NS, of long duration appears in about 4–28 % of cases more often in old age and prognosis is poor [30, 32]. Sarcoid changes can be located intra- or extramedullary (intra or extra meninges). According to Junger et al. [48] the spinal form occurs in four stages:

1. Leptomeningeal enhancement
2. Fusiform spinal cord enlargement
3. Focal or diffuse intramedullary disease
4. Spinal cord atrophy

**Fig. 3:**
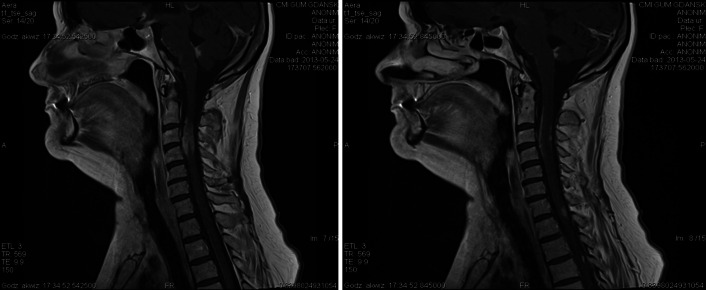
Neurosarcoidosis in MRI cervical spine in sagital plane: T1-weighted contrast-enhanced MR image shows enhancing leptomeningeal lesions involving of the spinal cord

Most frequently stages 2 and 3 occur in this form of NS, according to cited authors. In a recent case series of 29 patients with spinal cord neurosarcoidosis revealed that lesions were mostly intramedullary (in 81 %), although in 48 % involved the leptomeninges [49]. Spinal SA is mostly revealed by back pain of radicular type, weakness and paresthesias of limbs may also occur [32]. An MRI scan is useful, where the spinal NS appears as an enhancement, a thickening of leptomeninges, fusiform thickening of the spinal cord, most often in the thoracic level or cervical, affecting three or more spinal segments (Fig. 3) [45, 49].

## Peripheral nervous system SA (PNS)

Acute or chronic peripheral neuropathy occurs in about 2–40 % of patients with NS [30]. Patients with NS from the Caucasian population are confirmed as having a greater predisposition to PNS than Afro-Americans. The most common form is symmetrical axonal sensory motor polyneuropathy, rare is demyelinating form, focal mononeuropathy, multi focal, polyradiculopthy or vascular neuropathy [21].

In PNS, the large nerve fibers are often affected (large fiber neuropathy—LFN) appearing as a disturbance of proprioceptive sensation and vibration. At a microscopic level sarcoid granulomas locate themselves in the perineurium and epineurium, while the endoneurium is mostly spared. The most useful diagnostic test is an electroneurography with a typically low amplitude of muscle response—M during stimulation of the nerve. This form of neuropathy has a mostly acute or subacute course and usually is associated with a good prognosis.

Rarer with a poor prognosis is a small fiber neuropathy (SFN) with disturbed senses of pain and temperature as well as frequently changed autonomic functions. SFN involves the small thinly myelinated (A delta) and unmyelinated (C), and autonomic nerve fibers due to axon loss or oxidative stress [25]. A high risk of developing SFN is also associated with an increased frequency in the occurrence of HLA-DQB1*0602 antigen and/or non-HLA polymorphic genes encoded proinflammatory cytokines, particularly in patients with advanced disease process. It is symptomized by burning pain, paresthesia, restless legs syndrome, reduced perspiration, skin dryness and disturbed blood circulation [25]. In contrast to LFN, a biopsy of skin with reduced intraepidermal nerve fiber density is essential in diagnosing of SFN.

## Muscle sarcoidosis

In muscles, sarcoid granuloma may locate between nerve fibers. Muscle sarcoidsis is mostly diagnosed asymptomatically, however, about 1 % of patients present with side effects such as pain, weakened muscle or atrophy [30]. The course of this form can be acute or subacute. In the laboratory blood tests often confirm an increased level of muscle enzymes such as phosphocreatine kinase (CPK), AspAT, also hyperkalemia and an increased level of ACE (30–40 %), sIL-2R. In an electromyography test a myogenic pattern is characteristic. Imaging tests are also helpful e.g. scintigraphy with Gal and FDG-PET. However, a biopsy from the affected muscle is the best diagnostic test.

## NS treatment

At present there are no set standards for the treatment of patients suffering from NS [1, 20–23, 30, 36, 49–61]. Most treatment recommendations derive from expert opinion from centers seeing several dozen cases annually, from case reports or from small uncontrolled case series [reviewed in 30, 36, 57]. The majority of authors recommend early symptomatic treatment of patients. Nozaki and Judson [36] suggested that specific treatment for NS needs to be given over 6 months and tapering of medications should be individualized based on the seriousness of the illness, response to the treatment, and toxicity of therapy. Relapses are common and may occur in 20–50 % cases following discontinuation of the treatment. Thus, many patients with recurrent relapses need treatment for prolonged periods, sometimes for years [59].

## Pharmacologic treatment

- Corticosteroids are considered the drug of choice for the treatment of sarcoidosis with monitoring for potential adverse events, e.g. peptic ulcer diseases, systemic fungal infection, active tuberculosis, hypersensitivity, cataract formation, diabetes mellitus or osteoporosis [reviewed in 1, 20–23, 30, 36, 51, 53, 59]:
  - The cranial neuropathy: according to the latest treatment algorithm for NS, proposed by Nozaki et al. (with input from personal communication from Professors Barney Stern and Robert P. Baughman) [36] in 2013, in the case of cranial neuropathy, particularly where nerve VII is affected, prednisone is recommended in a daily dose of 20-40 mg with a decrease of dosage over 1–6 months to the lowest effective dose; if corticosteroids cannot be tapered to less than 10 mg/day of prednisone equivalent within 3–6 months, consideration should be given to higher dose prednisone and/or alternative agents [51]; there was a tendency for recurrence of symptoms at doses of prednisone less than 20–25 mg/day [reviewed in 1, 36, 51].
  - The mild or moderate NS, usually initiate with 20–60 mg/day (prednisone or prednisone equivalent) with a decrease of dosage to the lowest effective dose; if corticosteroids cannot be tapered to less than 10 mg/day of prednisone equivalent within 3–6 months, consideration should be given to higher dose prednisone and/or alternative agents [reviewed in 20, 36].
  - The severe NS (e.g. altered sensorium, visual loss, or weakness), cases not responding to oral agents, and refractory NS: the administration of intravenous methylprednisolone is advised, at 1 g daily for 3–5 days followed by 1 g per week or daily prednisone orally with a decrease of dosage to the lowest effective dose [reviewed in 36].
- Immunomodulating and cytotoxic agents such as methotrexate, cyclosporine, cyclophosphamide, or azathioprine
  - Methotrexate (MTX)—is used as a first-line corticosteroid-sparing agent and allows tapering of prednisone dose to 10 or 20 mg/day in more than one-third of NS cases [reviewed in 36, 51]; it is also reported a beneficial response to MTX in approximately one-fifth of NS patients, who failed prednisone monotherapy [52]; a combination of corticosteroids and MTX has resulted in a favorable outcome in patients with severe CNS involvement [reviewed in 36]; the effectiveness of MTX, given orally, mostly in an initial dose of 7.5 mg/per week and then 10–20 mg/week is noted in about 60 % of NS patients [27]; it requires monitoring for adverse events, e.g. hepatotoxicity, renal insufficiency, pneumonitis, teratogenicity, bone marrow suppression; an administration of folinic acid may reduce toxicity [reviewed in 36].
  - Azathioprine—has been used for corticosteroid-refractory NS with potential adverse effects, like neutropenia, abnormal liver function tests, pancreatitis or allergic reactions [reviewed in 30, 55–57]; usual oral dose of azathioprine is 2–3 mg/kg [reviewed in 57].
  - Cyclosporine—it was beneficial in some patients, who failed prednisone monotherapy; others worsened in spite of a combination of cyclosporine and corticosteroid therapy [28]; it can be started at 4 mg/kg/day in divided doses and requires monitoring for adverse events, e.g. hypertension, renal failure, hypomagnesemia, and neurotoxicity [reviewed in 30].
  - Cyclophosphamide—due to its high toxicity and significant side effects (bone marrow suppression, teratogenicity and carcinogenicity), the use of it is usually limited to severe NS, when TNF-α antagonist cannot be obtained, in NS refractory to other agents; it is also corticosteroid-sparing agent in NS and allows tapering of prednisone dose to 10 mg [52, 53]; according to authors [53, 54], dose of cyclophosphamide is 500–1000 mg intravenously over 30–60 min every 2–4 weeks or 0.5 g/m^2^ of body surface area intravenously every 4 weeks depending on age, leukocyte count or renal function; given orally, an initial dose 25–50 mg/day is increased by 25 mg increments to ensure adequate immunosuppression; the maximum oral dose is 150 mg/day; intravenous administration of cyclophosphamide is preferred over oral administration due to a lesser side effects [54].
  - Mycophenalate mofetil—immunosuppressant as an analog of methotrexate is considered for patients with central NS, though not effective in NS of the muscles [reviewed in 36].
  - Anti-malarial agents, like chloroquine and hydroxychloroquine have anti-inflammatory effects and they are also effective in sarcoidosis, especially in patients with NS and sarcoidosis-induced hypercalcemia and/or sarcoid skin lesions; its standard oral dosage is 250–750 mg daily; hydroxychloroquine has lower risk of ocular toxicity than chloroquine, but is less effective than chloroquine; because hydroxychloroquine reduces serum glucose levels, it may be useful for NS patients with steroid-induced hyperglycemia; its standard dosage is 200–400 mg daily orally [20, 21, 36, 57, 59].
  - A tumor necrosis factor alpha (TNF-α) inhibitor—e.g. infliximab, adalimumab, thalidomide, pentoxifylline: infliximab is commonly used for neurosarcoidosis that is refractory to other agents and/or corticosteroid-induced complications; in a recent case series of 29 patients with spinal cord neurosarcoidosis, infliximab was the second most frequently used immunosuppressive agent after corticosteroids [49]; it is also useful for therapy of SFN [30, 50, 51]; infliximab 3–5 mg/kg intravenously every 4–8 weeks; often, patients receive an initial loading dose of 3–5 mg/kg intravenously at weeks 0, 2, and 6 [reviewed in 36]; adalimumab and teratogenic thalidomide are effective especially for spinal forms of NS [30, 36, 50, 51, 58]; pentoxifylline have been shown to be effective for various types of organ involvement from systemic sarcoidosis, however, their role in the treatment of neurosarcoidosis is unknown [reviewed in 36, 57]; several major toxicities are encountered with the anti-TNF therapies, but one of the more serious concerns is the increased risk of tuberculosis and similar infections [36, 49, 57].
- Treatment of associated conditions

In addition to specific treatment for sarcoidosis, treatment for associated conditions (e.g., anti-epileptics, medicines for neuropathic pain or hormonal replacement therapy in a neuroendocrine dysfunction) may be required for the treatment of neurosarcoidosis [20, 21, 30, 36, 50, 51].

## Neurosurgical treatment

Patients with NS, who qualify for neurosurgery are those with hydrocephalus (a ventricle-peritoneum shunt is inserted), pseudotumor forms with the mass effect in the brain and spinal cord (resection of changes) [36].

## Radiotherapy

In the case of a lack of response to pharmacological treatment or a contraindication to surgical procedure, a cerebral radiotherapy is also recommended [reviewed in 30, 36, 61, 62]. Bruns et al. [61] revealed a partial resolution of the clinical features and stabilization of NS after low-dose whole-brain irradiation of the isolated CNS lesion with 20 Gy. Also Chapelon et al. [62] introduced a patient with sarcoid meningitis, who was sensitive to radiotherapy and recovered after 200 rads, whereas NS patients with hemiparesis, an extrapyramidal syndrome, and severe psychiatric features unresponsive to steroids, improved after 6,000 rads. Radiotherapy (1.3–3.6 Gy/day for 3–24 weeks) was also beneficial in one of the three patients treated by Agbogu et al. [53].
